# Heligmosomid infections in bank voles are associated with higher prevalence and greater abundance of other helminth species

**DOI:** 10.1017/S0031182025101376

**Published:** 2026-02

**Authors:** Jerzy M. Behnke, Joseph A. Jackson, Anna Bajer, Mohammed Alsarraf, Jolanta Behnke-Borowczyk, Maciej Grzybek

**Affiliations:** 1 School of Life Sciences, University of Nottingham, University Park, Nottingham, UK; 2 School of Science, Engineering and Environment, University of Salford, Manchester, UK; 3 Department of Eco-Epidemiology of Parasitic Diseases, Institute of Developmental Biology and Biomedical Sciences, Faculty of Biology, University of Warsaw, Warsaw, Poland; 4 Faculty of Forestry and Wood Technology, Poznań University of Life Sciences, Poznań, Poland; 5 Institute of Maritime and Tropical Medicine, Medical University of Gdańsk, Gdynia, Poland

**Keywords:** abundance, associations, bank voles, *Clethrionomys glareolus*, coinfection, *Heligmosomoides glareoli*, *Heligmosomum mixtum*, *Myodes glareolus*, positive covariance, prevalence

## Abstract

The heligmosomid nematodes *Heligmosomum mixtum* and *Heligmosomoides glareoli* are dominant helminths infecting bank voles (*Clethrionomys glareolus*) in the temperate forests of NE Poland. Both are relatively long-lived species that accumulate in hosts with increasing host age. Based on studies showing that the closely related species, *Heligmosomoides bakeri* is immunomodulatory in murine hosts, we hypothesized that heligmosomid-infected bank voles should show higher prevalence and abundance with other helminths. To test this hypothesis, we analysed a database containing quantitative data on helminth parasites of bank voles (*n* = 922), comprising worm burdens recorded during 4 surveys, conducted at 3- to 4-year intervals, in 3 forest sites, during late summer of each year. After controlling for both intrinsic and extrinsic factors, the presence of heligmosomid nematodes was significantly associated with higher species richness of other helminth species, with the greater likelihood of voles carrying other helminth species, with higher worm burdens of other helminths and with significant positive covariance of heligmosomid burdens with those of other concurrently residing helminths. These patterns might be explained by a number of biological processes, including correlated host exposure or correlated host susceptibility not driven by the parasitic infections themselves. However, we consider it most likely that these results are consistent with the idea that like *H. bakeri*, the heligmosomid nematodes of bank voles employ non-specific immunomodulation to facilitate their own long-term survival, with the consequence that other concurrently infecting intestinal helminths benefit.

## Graphical abstract

**Figure:**
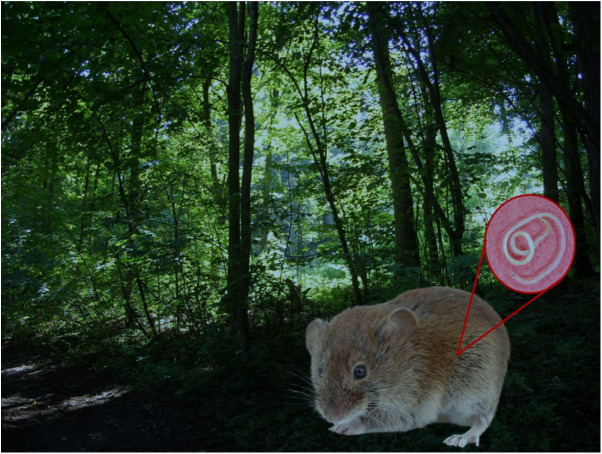

## Introduction

Interactions between free-living species are the hallmarks of animal and plant communities (Gotelli and Graves, 1996; Morin, 2011). Positive interactions reflect symbiotic and mutualistic relationships, as for example among nectar-feeding insects pollinating flowers, while negative interactions in which the abundance of 1 species is reduced in the presence of another, may arise through predation as part of the food chain or through competition for limited resources (Morin, 2011).

Similarly, in disease ecology interactions between different parasite species in hosts may be either positive or negative (Montgomery and Montgomery, 1990; Haukisalmi and Henttonen, 1998). The latter arise because for 1 or more reasons the host environment is made less suitable for the second species when the first is present. In consequence, the second species may be less likely to establish in the host, its growth and development may be delayed or impaired entirely, it may be excluded from the optimum site for its development in the host and/or its longevity may be curtailed in hosts concurrently infected with the first species. The underlying mechanisms, often based on cross immunity, generation of non-specific inflammatory responses, parasite-induced host mortality or competition for resources in concurrently infected hosts, have been reviewed comprehensively in earlier publications (Cox, 2001; Behnke et al. 2001a; Pedersen and Fenton, 2007; Cattadori et al. 2008).

Positive interactions among infectious agents, resulting in co-occurrence in hosts, may arise because the first species modifies the host’s internal environment in a way that improves the establishment, development and survival of the second species. The most likely such mechanism is through depression of the host’s defences by the first species (Pritchard and Behnke, 1985; Hewitson et al., 2011; Maizels et al., 2012), as part of its survival strategy, with the consequent knock-on effect of also benefiting the survival of other co-infecting species (Behnke et al., 1978, 1984, 2009).

Interactions among parasites are an important interest because they affect the way parasite communities assemble and in turn, the progression of disease within individual hosts and the onward transmission of infection between hosts (Graham et al., 2007; Bradley and Jackson, 2008). Through acting as regulatory feedbacks, interactions could even affect the long-term stability of host–parasite assemblages (Jackson et al., 2023). Positive and negative interactions between parasites might sometimes be detectable as statistical associations in count or prevalence data from surveys of free-running systems. Unfortunately, however, a number of coincidental processes may produce such statistical associations, confounding the ability to detect true interactions that could act as genuine regulatory feedbacks (Bradley and Jackson, 2008; Barger, 2023). Such coincidental processes result from correlated susceptibility or correlated exposure (Bradley and Jackson, 2008). Correlated exposure may arise because the infective stages of multiple species co-locate in proximity in the same external environment as that resided in by the host and concurrent transmission is therefore likely. For example, the definitive host of multiple parasite species may feed on an animal utilized by all of these parasites as an intermediate host for their larval stages or on one that acts as a paratenic host for the parasites concerned (Behnke et al., 2024). Correlated susceptibility may occur because of genetic variation in the host that simultaneously affects resistance to multiple species, or because of environmental factors that affect host quality and the general ability of hosts to defend themselves against parasitic infection.

Parasite–parasite interactions have been demonstrated clearly many times in laboratory systems (Holmes, 1961; Cox, 2001; Behnke et al., 2001a) but the evidence for their importance in more complex real-world situations, where infection exposures and host phenotypes may be very different to those in the laboratory, remains somewhat contradictory (Poulin, 2001; Telfer et al., 2008, 2010: Knowles et al., 2013; Keegan et al., 2024) and an active area of research. While field-scale experiments (Knowles et al., 2013; Fenton et al., 2014) or analysis of individual time series (longitudinal study designs) (Telfer et al., 2008) provide a potentially powerful mode of inference in natural systems, that could partition genuine interactions from other processes and reveal their true sign, nonetheless they are also subject to very significant practical and technical difficulties. Thus, large-scale observational surveys (cross-sectional study designs), that are easier to execute and replicate, and that search for evidence of an interaction through statistical association in species counts or prevalence, are still relevant. In particular, consistent patterns of association, linked to individual taxa and predictable from the known biological propensities of those taxa, might still be taken as important corroborating evidence supporting the existence of interactions in the real world. Here, we present such an example, based on a very large survey of a community that included species from a group with known immunosuppressive properties.

Nematode parasites of the family Heligmosomidae are parasites of rodents, and 1 species in particular, *Heligmosomoides bakeri* has been shown through extensive laboratory based experimental infections to give rise to chronic infections (Robinson et al., 1989) and to be immunomodulatory in its murine host as part of its survival strategy in the face of the adaptive immune system (Behnke et al., 1983; Hewitson et al., 2011; Maizels et al., 2012; Smith et al., 2016; Filbey et al., 2020). A possible consequence of such a strategy is enablement of longer survival of other concurrently resident species (Jenkins and Behnke, 1977; Behnke et al., 1978, 1984). The mechanism of immunodepression is complex and has been reviewed (Maizels et al., 2012, 2018; Shepherd et al., 2015; Yap and Gause, 2018; McManus and Maizels, 2023). Significantly, *H. bakeri*’s closely related congeneric species *H. polygyrus* has been shown to accumulate with age in naturally occurring *Apodemus sylvaticus* hosts (Gregory, 1992; Gregory et al., 1992; Behnke et al., 1999; Friberg et al., 2013; Loxton et al., 2017). The occurrence of *H. polygyrus* is furthermore associated with increasing species richness of other helminth species under natural conditions (Behnke et al., 2005, 2009), although antagonistic (negative) interactions with coccidia (Protozoa) have also been documented in field experiments (Knowles et al., 2013). Apart from these studies on *H. polygyrus*, little is known about whether other naturally occurring heligmosomid nematodes employ immunodepression for survival in definitive hosts.

Two heligmosomid nematodes, *Heligmosomoides glareoli* and *Heligmosomum mixtum* are dominant helminths of bank voles (*C. glareolus;* Haukisalmi and Henttonen, 1993a, 1993b) in continental Europe. The prevalence of both species in bank voles may be high, while abundance seldom exceeds 10 worms/host (Haukisalmi, 1986; Haukisalmi and Henttonen, 1993a; Grzybek et al., 2015). Like *H. polygyrus*, both accumulate in their hosts with increasing host age (Grzybek et al., 2015), suggesting that they give rise to chronic infections. If these species, like their congeners modify the host environment to benefit their own long-term survival, then we may expect the prevalence and abundance of other helminth species, as well as helminth species richness to be enhanced in heligmosomid-infected bank voles. To test this prediction we analysed the database, utilized by Grzybek et al. (2015), which comprises over 900 bank vole records of animals sampled at 3- to 4-year intervals over more than a decade in 3 disparate sites in NE Poland, specifically seeking evidence for a positive association between the heligmosomids and other helminth taxa.

## Materials and methods

### Study sites and source of data

The following analysis utilized the database of Grzybek et al. (2015). These authors reported on the long-term spatiotemporal stability and changes in the helminth communities of bank voles from 3 study sites (Urwitałt, Tałty and Pilchy) in the temperate forests of NE Poland, named after nearby villages. The animals were trapped during 4 surveys conducted initially at 3, then 4-year intervals (1999, 2002, 2006 and 2010) in late summer (August–September) in each of these years. Descriptions of the sites, the methodology used in fieldwork and for subsequent laboratory analysis of worm burdens have all been described comprehensively in earlier publications (Behnke et al., 2001b, 2008a, 2008b; Grzybek et al., 2015). Based on morphometric data collected at autopsy and dried eye lens weight, the animals were allocated to 3 age classes (age classes 1, 2 and 3, corresponding to immature, young, adult and mature older voles, respectively; see Behnke et al., 2001b) and were sexed (male or female).

### Definition of terms employed

In this paper, we refer to *Clethrionomys glareolus* (as recommended by the Mammal Diversity Database; Upham et al., 2023; Tesakov et al., 2010; Kryštufek et al., 2020), rather than *Myodes glareolus* (see Carleton et al., 2014), following several recent revisions of the generic attribution of bank voles.

We focus on the effect of 2 species of heligmosomid nematodes (Heligmosomidae; *Heligmosomoides glareoli* and *Heligmosomum mixtum*) on other helminth taxa. These 2 species were treated as 1 taxon and are referred to hereafter as the heligmosomids. We refer also to *H. bakeri*, a parasite of *Mu*s spp., previously known as *H. polygyrus* and *Nematospiroides dubius* (Behnke et al., 1991; Cable et al., 2006). All other species of helminths were pooled into the following groups for analysis:

1. Non-heligmosomid helminths (all other helminth species, referred to also as other helminths),
2. Non-heligmosomid nematodes (all other nematode species, referred to also as other nematodes),
3. Oxyuroidea (*Aspiculuris tianjinensis* and *Syphacia* spp.),
4. Cestodes (all cestode species and individual worms, both immature and mature intestinal strobilate stages and tissue dwelling larvae),
5. Intestinal cestodes (adult, strobilate stages from intestines) and
6. Larval cestodes (all larval stages, found in the pleural and peritoneal cavities, and associated organs).

For further details of all the species involved, see Table 1.

**Table 1.** The overall prevalence and abundance of helminths recovered from *Clethrionomys glareolus* in 3 study sites in NE Poland during 4 surveys at 3- to 4-year intervals (*n* = 922)

		Prevalence		Abundance	
Taxon	Species	% Infected	CL_95_	Mean ±	SEM
Helminths (all species)		79.7	76.12−82.92	26.6	5.68
Nematoda (all species)		77.0	73.30−80.36	21.4	5.52
Heligmosomidae (both species)		55.5	51.34−59.68	2.3	0.12
	*Heligmosomum mixtum*	37.6	33.62−41.81	1.4	0.08
	*Heligmosomoides glareoli*	19.0	15.90−22.48	0.89	0.101
Non-heligmosomid helminths		62.9	58.73−66.92	24.4	5.68
Non-heligmosomid nematodes		54.6	50.36−58.75	19.2	5.52
Oxyuroidea (both species)		45.0	40.81−49.20	17.7	5.51
	*Aspiculuris tianjinensis*	42.1	37.98−46.27	7.2	0.80
	*Syphacia petrusewiczi*	3.3	2.02−5.08	10.4	5.47
Other nematode species					
	*Mastophorus muris*	14.3	11.59−17.52	0.70	0.099
	*Aonchotheca annulosa*	6.4	4.58−8.78	0.78	0.243
	*Trichuris arvicolae*	0.4	0.18−1.38	0.0065	0.00343
Cestoda (all species)		20.6	17.37−24.21	5.2	1.39
Cestoda, intestinal stages		16.3	13.36−19.61	0.34	0.040
	*Catenotaenia henttoneni*	15.0	12.18−18.18	0.32	0.039
	*Paranoplocephala kalelai*	0.7	0.27−1.72	0.0065	0.0026
	*Arostrilepis horrida*	0.1	0.05−0.86	0.0011	0.00109
Cestoda, larval stages		6.9	5.03−9.38	4.9	1.39
	*Mesocestoides* spp.	3.6	2.29−5.47	4.2	1.34
	*Taenia martis*	2.1	1.15−3.64	0.029	0.0092
	*Versteria mustelae*	1.4	0.07−2.80	0.037	0.0157
	*Cladotaenia globifera*	0.3	0.14−1.20	0.60	0.363

Most of the data in this table were included in the text in Grzybek et al. (2015).

### Statistical analysis

For data subsets, prevalence values (% of voles infected with the helminth taxon referred to) are given with 95% exact confidence limits (CL_95_) in the tables and text, and 95% confidence intervals (CI_95_) are illustrated in the figures. These were calculated in bespoke software based on the tables of Rohlf and Sokal (1995). For quantitative data (i.e. abundance as reflected in worm burdens), mean values ± standard error of the mean (SEM) are provided and these refer to abundance as defined by Bush et al. (1997) and Margolis et al. (1982) and are derived for all voles in a data subset, including those uninfected by the worms of the relevant taxon.

The frequency distributions of worm burdens of all the taxa employed for analysis were tested by *x*^2^ for goodness of fit to positive binomial, negative binomial (negbin), Poisson and Gaussian distributions as relevant. We provide values for the index of dispersion (mean/variance ratio, *I*), index of discrepancy (*D*; Poulin, 1993), and the negative binomial exponent (*k*) for each of the heligmosomid taxa separately and when combined (Figure S1).

For consistency with our previous work (Grzybek et al., 2015), prevalences were analysed using maximum likelihood techniques based on log linear analysis of contingency tables in the software package IBM SPSS, version 28 (Armonk, New York, USA). Full factorial models included SEX (2 levels, male and female voles), AGE (3 levels, juvenile, young adult and fully mature voles), geographical locality, i.e. SITE (3 levels corresponding to the 3 study sites as specified above), YEAR of survey (1999, 2002, 2006 and 2010), and these are referred to as the intrinsic (sex and age class) and extrinsic (site and year) factors. Models also included presence/absence (P/A) of infection with the heligmosomid worms. This approach is based on categorical values of factors of interest, which are used not only to fit hierarchical log-linear models to multidimensional cross-tabulations using an iterative proportional-fitting algorithm but also to detect associations between the fitted factors. Multi-factorial models were fitted as described previously (Grzybek et al., 2015), beginning with the full factorial model, followed by simplification of the model until only significant terms remained. Thus, for each level of analysis in turn, beginning with the most complex model, involving all possible main effects and interactions, those combinations that did not contribute significantly to explaining variation in the data were eliminated in a stepwise fashion, beginning with the highest-level interaction (backward selection procedure). A minimum sufficient model (MSM) was then obtained, for which the likelihood ratio of Chi-squared was not significant, indicating that the model was sufficient in explaining the data. The importance of each term in interactions involving P/A of a specified taxon in the final model was assessed by the probability that its exclusion would alter the model significantly and these values are given in the text, assessed by a likelihood ratio test between models with and without each term of interest.

For analysis of quantitative data, we first fitted full factorial (FFM) generalized linear (GLM) statistical models in R (v. 4.3.2, R Core Development Team) with both extrinsic (YEAR and SITE) and intrinsic (SEX and AGE) fixed factors for all dependent variables, testing models that were based on negative binomial or Poisson error structures, as relevant. The dependent variables were worm burdens of the groups listed in Table 2 and helminth species richness of all taxa other than the heligmosomids. Wherever possible, we used the ‘step’ command in R to refine these to minimum sufficient models (MSMs). However, in some cases, full factorial GLMs could not be simplified, and in others only reduced models (best-fit model, BFMs) would converge appropriately, and in this latter case, we specify in the text or in parenthesis the factors that were successfully fitted. Next, we combined the FFMs, MSMs, or BFMs with the addition of P/A of heligmosomid worms as a factor at 2 levels (present or absent). To evaluate whether worm burdens of other taxa differed significantly between voles with/without heligmosomids we compared models with and without (P/A) heligmosomids.

**Table 2.** Prevalence and abundance of non-heligmosomid helminth (i.e. Not Heligmosomidae) species and higher taxa in bank voles with and without heligmosomids. Data have been pooled, and include all years and sites, and both host sex and age classes

	Prevalence				Abundance			
	Heligmosomids present (*n* = 512)		Heligmosomids absent (*n* = 410)		Heligmosomids present (*n* = 512)		Heligmosomids absent (*n* = 410)	
	%	CL_95_	%	CL_95_	Mean	SEM	Mean	SEM
Non-heligmosomid helminths	**69.7**	66.76−72.55	54.4	48.17−60.55	**35.2**	10.08	10.8	2.04
Non-heligmosomid nematodes	**59.6**	56.45−62.62	48.3	42.07−54.51	**26.5**	9.80	9.9	2.01
Oxyuroidea combined	**48.0**	44.92−51.18	41.2	35.19−47.43	**24.5**	9.79	9.1	2.01
*Aspiculuris tianjinensis*	**45.1**	41.99−48.24	38.3	32.47−44.50	**8.4**	1.25	5.7	0.91
*Syphacia petrusewiczi*	**3.3**	2.35−4.66	3.2	1.56−6.16	**16.1**	9.74	3.4	1.81
*Mastophorus muris*	**18.2**	15.82−20.73	9.5	6.40−13.84	**0.81**	0.131	0.57	0.150
*Aonchotheca annulosa*	**8.2**	6.61−10.08	4.1	2.20−7.38	**1.23**	0.426	0.22	0.118
Cestodes, all stages	**27.1**	24.42−30.03	12.4	8.84−17.12	**8.67**	2.475	0.86	0.319
Cestodes, intestinal stages	**20.9**	18.42−23.56	10.5	7.15−14.93	**0.43**	0.060	0.22	0.047
*Catenotaenia henttoneni*	**19.3**	16.94−21.92	9.5	6.40−13.84	**0.41**	0.060	0.21	0.047
Cestodes, larval stages	**10.0**	8.19−12.01	3.2	1.56−6.16	**8.24**	2.473	0.64	0.316

The higher prevalences and abundances at each level are highlighted in bold.

To test whether increasing worm burdens of heligmosomids were associated with increasing worm burdens of specified taxa (i.e. whether there was quantitative covariance between the worm burdens of these taxa), we combined models of those taxa, as fitted above, with the (deviance) residuals of the MSM for heligmosomid worm burdens but fitted as a covariate. This approach removes correlation between heligmosomid worm burdens and the other explanatory variables and is conservative (i.e. only assigning variance explained by the heligmosomid count variable over and above that already explained by other correlated explanatory variables). Furthermore, effects from these models could be visualized on the scale of the response worm burdens. We also examined in R, linear covariance of the residuals of FFMs, MSMs or BFMs, as relevant, of specified taxa with the residuals of the MSM for heligmosomid worms and then tested whether slopes (*ß* ± SEM) were positive and whether they differed significantly from zero. This approach not only deals with correlation between the explanatory variable of interest and other explanatory variables (as above) conservatively, but also preserves the maximum amount of degrees of freedom for the key test of the hypothesis. In all these cases, we predicted positive slopes, and hence we employed 1-tailed values for *P*. Figures of the resulting relationships were constructed in the *ggplot2* and *sjPlot* packages of R. Values of likelihood ratios (*LR*) for models based on negative binomial errors and deviances (*Dev*) for models with Poisson errors are provided.

For analysis of ordinal data (e.g. age class) we used the Mann–Whitney *U* test in the software package IBM SPSS, version 28.

The covariance distribution test of Haukisalmi and Henttonen (1998), which focuses on the frequency distribution of pairwise covariances (i.e. positive and negative covariances [cov] between all pairs of species in a community), was applied to the data. We used both log_10_ + 1 and square root transformations of worm counts, and then obtained significance values from assessment of the number of times the observed covariance for each pair of species was less than or greater than that derived from each of 5000 cycles of randomization of the data. The test was then repeated with subsample constraints, i.e. with year, site, and host age and sex, taken into consideration.

## Results

### Age of voles that were infected or not infected with heligmosomid nematodes

Helminths are known to accumulate in hosts exposed to natural infections, such that worm burdens and helminth species diversity both increase with host age (Montgomery and Montgomery, 1989; Behnke et al., 1999; Grzybek et al., 2015). Therefore, it was necessary to determine first whether the 2 key groups in this study (voles infected with heligmosomid nematodes and those without heligmosomids) differed in age. Since the voles had been allocated to 3 age classes (1, 2 or 3) based on a range of morphological features, we averaged the age classes. Voles without heligmosomids had an average age class of 1.81 ± 0.039, while those infected with heligmosomids 2.22 ± 0.034. This difference is significant (Mann–Whitney *U* test, *z* = 7.754, *P* < 0.001).

### Overall prevalence and abundance of helminths in bank voles

Table 1 gives the overall prevalence and mean worm burdens of the helminths recovered from *C. glareolus*, with data combined across the 3 study sites, 4 surveys, both sexes and the 3 age classes. Collectively, nematodes accounted for most of the helminths recovered (80.5% of all the helminths recovered were nematodes). For a breakdown of these data by the 4 factors and relevant analyses, see Grzybek et al. (2015).

Across the whole study period the most prevalent species was *A. tianjinensis* (42.1%), and individually the 2 heligmosomid species were the next most prevalent, but when combined their prevalence was 55.5%. The frequency distributions of worm burdens with *H. mixtum, H. glareoli* and the 2 heligmosomids combined are shown in Fig. S1. For each taxon the best-fit distribution was the negative binomial: for *H. mixtum* (*χ*^2^_11_ = 14.1, *P* = 0.229); *H. glareoli* (*χ*^2^_8_ = 16.9, *P* = 0.031); heligmosomids combined (*χ*^2^_13_ = 16.3, *P* = 0.234).

### Does the presence of heligmosomids affect prevalence of other helminths?

**Figure 1.**
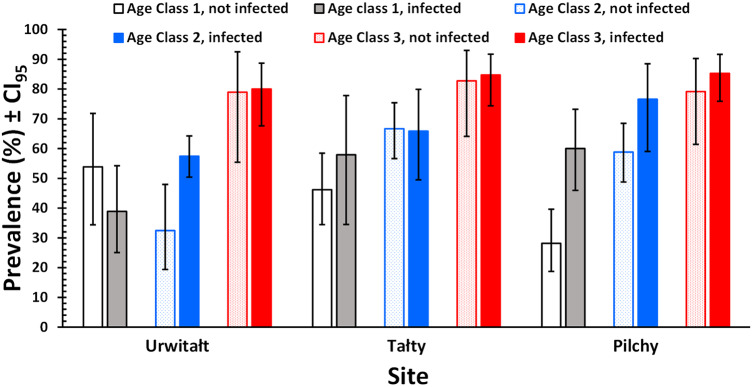
Prevalence of non-heligmosomid helminths in bank voles infected or not infected with heligmosomid nematodes, by year and site.

The prevalence of non-heligmosomid helminths was higher in bank voles with (69.7% [66.76–72.55%]), compared to those without heligmosomid nematodes (54.4% [48.17–60.55%]). In a model without extrinsic (YEAR and SITE) or intrinsic (AGE and SEX) factors, this difference was highly significant (*χ*^2^_1_ = 22.9, *P* < 0.001; Table 2). However, when all 4 factors were fitted, this relationship was dependent on both site and age of the voles (P/A heligmosomids × P/A other helminths × SITE × AGE CLASS, *χ*^2^_4_ = 11.3, *P* = 0.024), as illustrated in Figure 1. Thus, while prevalence of non-heligmosomid helminths was numerically higher overall in bank voles that carried heligmosomid nematodes in each major subset of data (i.e. by year, site, sex, and age; Table 3), there was a significant difference in how prevalence varied with host age between the 3 study sites. Although, it can be seen in Figure 1 that prevalence of non-heligmosomid helminths was mostly higher in voles also carrying heligmosomids in each of the 3 age classes, especially at Pilchy, the bias was in the opposite direction among age class 1 voles from Urwitałt and age class 2 voles from Tałty.

**Table 3.** Prevalence of non-heligmosomid helminths (i.e. not Heligmosomidae) and non-heligmosomid nematodes in bank voles with and without heligmosomids by year, site, host sex and age class

		Non-heligmosomid helminths						Non-heligmosomid nematodes			
		Heligmosomids present			Heligmosomids absent			Heligmosomids present		Heligmosomids absent	
Factor	Level	*n*	%	CL_95_	*n*	%	CL_95_	%	CL_95_	%	CL95
Year	1999	102	**57.8**	50.79−64.67	37	45.9	31.36−61.59	**46.1**	39.01−53.14	45.9	31.36−61.59
	2002	92	**82.6**	70.38−90.81	127	63.0	55.19−70.21	**65.2**	51.87−76.91	53.5	45.73−61.25
	2006	164	**76.8**	68.47−83.70	106	63.2	56.04−69.89	**71.3**	62.64−78.67	56.6	49.42−63.54
	2010	154	**62.3**	53.73−70.23	140	42.1	34.26−50.34	**52.6**	43.97−61.06	37.9	30.32−46.05
Site	Urwitałt	222	**63.1**	58.49−67.44	82	50.0	37.36−62.64	**46.4**	41.80−50.99	39.0	27.50−51.59
	Tałty	132	**75.0**	67.58−81.20	158	59.5	50.76−67.85	**67.4**	59.52−74.44	53.8	45.05−62.28
	Pilchy	158	**74.7**	66.49−81.51	170	51.8	42.65−60.80	**71.5**	63.05−78.71	47.6	38.71−56.76
Sex	Males	262	**66.4**	61.57−70.94	209	51.7	47.20−56.15	**54.6**	49.65−59.49	43.5	39.17−48.01
	Females	250	**73.2**	68.69−77.29	201	57.2	52.82−61.52	**64.8**	60.06−69.30	53.2	48.83−57.64
Age	Class 1	105	**52.4**	45.23−59.53	175	40.0	31.23−49.25	**40.6**	32.92−48.58	36.7	29.01−45.13
	Class 2	189	**64.0**	54.37−72.82	139	54.7	46.50−62.57	**51.5**	43.63−59.43	50.0	40.03−59.97
	Class 3	218	**83.0**	79.31−86.25	96	80.2	66.85−89.29	**78.7**	69.99−85.63	64.3	56.53−71.46i

The higher prevalence at each level is highlighted in bold.

### Does the presence of heligmosomids affect prevalence of other nematodes?

A similar picture emerged when the analysis was restricted to non-heligmosomid nematodes. Prevalence of non-heligmosomid nematodes was higher overall in bank voles with (59.6% [56.45–62.62%]) compared with those without heligmosomid nematodes (48.3% [42.07–54.51%]), and in each of the data subsets (Tables 2 and 3). In a model without extrinsic (YEAR and SITE) or intrinsic (AGE and SEX) factors, this difference was highly significant (*χ*^2^_1_ = 11.7, *P* < 0.001) and retained independent significance when all 4 factors and their interactions were taken into consideration (P/A heligmosomids × P/A other nematodes, *χ*^2^_1_ = 4.69, *P* = 0.030).

Restricting the analysis further to the Oxyuroidea (Table 2), yielded a weaker relationship (P/A heligmosomids × P/A Oxyuroidea, *χ*^2^_1_ = 4.30, *P* = 0.038) when the 4 factors were not included in the model, but lost significance when the 4 factors were taken into account (*χ*^2^_1_ = 3.45, *P* = 0.063). We found a similar outcome for *A. annulosa*, with a significant difference when no factors were included (*χ*^2^_1_ = 6.51, *P* = 0.011; Table 2) but loss of significance when we controlled for both intrinsic and extrinsic factors (*χ*^2^_1_ = 0.52, *P* = 0.47). Likewise, the overall prevalence of *M. muris* was also numerically higher in voles with heligmosomid nematodes compared to those without and this difference was highly significant (*χ*^2^_1_ = 14.4, *P* < 0.001; Table 2). However, in this case inclusion of the 4 factors in the model yielded 2 significant but complex terms (P/A heligmosomids × P/A *M. muris* × YEAR × SITE × SEX, *χ*^2^_6_ = 15.5, *P* = 0.017; P/A heligmosomids × P/A *M. muris* × SITE × SEX × AGE, *χ*^2^_4_ = 20.2, *P* < 0.001), which were not explored further.

### Does the presence of heligmosomids affect prevalence of cestodes?

**Figure 2.**
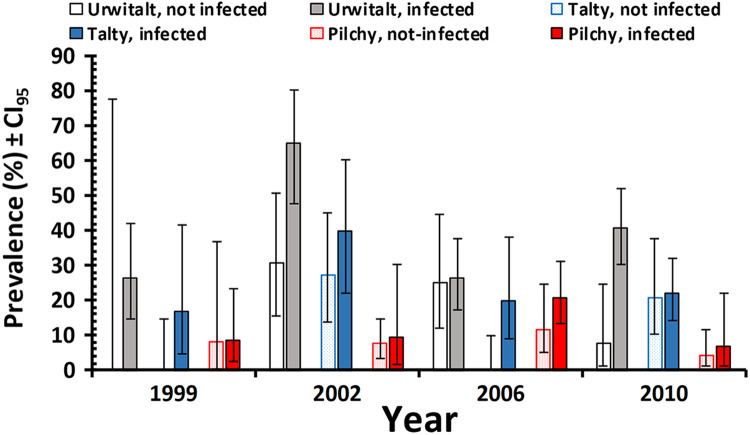
Prevalence of cestodes (all species and stages combined) in bank voles infected or not infected with heligmosomid nematodes, by age class and site.

In a model without the 4 factors, the prevalence of cestodes (all stages combined) was significantly higher among voles infected with heligmosomid nematodes (Table 2; *χ*^2^_1_ = 31.3, *P* < 0.001). This retained significance when the 4 factors were taken into account but was confounded by interactions with YEAR and SITE (P/A heligmosomids x P/A cestodes × SITE × YEAR, *χ*^2^_6_ = 17.8, *P* = 0.007). This interaction arose because while the difference between prevalence in heligmosomid infected and not-infected voles was always biased in favour of the former (in all 12 data subsets illustrated in Figure 2), the extent of the difference varied between years and sites.

Prevalence of intestinal, strobilate stages of cestodes was significantly higher in voles infected with heligmosomid nematodes (Table 2), and independent of all 4 factors and interactions between them (P/A heligmosomids x P/A intestinal cestodes, *χ*^2^_1_ = 4.22, *P* = 0.04). The bias in favour of higher prevalence of intestinal cestodes among heligmosomid-infected voles was evident in each year and site, and in both sexes and all 3 age classes (Table 4). A similar model with larval cestode stages revealed that there was also a bias for higher prevalence among heligmosomid-infected animals, but that it was confounded by host sex (P/A heligmosomids × P/A larval cestodes × SEX, *χ*^2^_1_ = 5.52, *P* = 0.019). While prevalence of larval cestodes was higher in heligmosomid-infected voles in each year, site, sex and age class (Table 4), in the case of sex, prevalence was 1.9 times higher in heligmosomid-infected male voles, but the disparity was much greater in female voles (7.2 higher in heligmosomid-infected voles; Table 4).

**Table 4.** Prevalence of intestinal strobilate and larval cestodes in bank voles with and without heligmosomids by year, site, host sex and age class

		Intestinal, strobilate cestodes				Larval stages of cestodes			
		Heligmosomids present		Heligmosomids absent		Heligmosomids present		Heligmosomids absent	
Factor	Level	%	CL_95_	%	CL_95_	%	CL_95_	%	CL_95_
Year	1999	**11.8**	7.82−17.01	0	0−8.77	**5.9**	3.31−10.08	2.7	0.26−13.38
	2002	**41.3**	28.71−54.69	15.7	10.79−22.18	**16.3**	8.17−28.46	6.3	3.42−11.17
	2006	**20.7**	14.28−28.69	9.4	5.94−14.51	**3.7**	1.37−8.59	0.9	0.16−3.64
	2010	**14.9**	9.64−22.08	9.3	5.46−15.19	**15.6**	10.21−22.75	2.1	0.64−6.16
Site	Urwitałt	**29.7**	25.68−34.10	18.3	10.36−29.83	**16.2**	13.06−19.88	6.1	2.08−15.26
	Tałty	**19.7**	14.03−26.77	12.7	7.77−19.49	**6.8**	3.76−11.97	2.5	0.78−7.00
	Pilchy	**9.5**	5.42−15.85	4.7	2.01−10.20	**3.8**	1.48−8.66	2.4	0.64−7.03
Sex	Males	**23.7**	19.68−28.13	12.4	9.74−15.72	**9.2**	6.67−12.47	4.8	3.18−7.06
	Females	**18.0**	14.52−22.06	8.5	6.28−11.28	**10.8**	8.08−14.20	1.5	0.73−3.00
Age	Class 1	**11.4**	7.48−16.75	7.4	3.76−13.86	**1.9**	0.63−5.10	0.6	0.05−4.21
	Class 2	**16.9**	10.77−25.48	10.1	6.05−16.00	**5.8**	2.62−11.95	2.9	1.06−7.02
	Class 3	**28.9**	24.91−33.22	16.7	8.33−29.18	**17.4**	14.17−21.19	8.3	3.07−19.00

The higher prevalence at each level is highlighted in bold. Sample sizes are as in Table 3.

### Does the presence of heligmosomid nematodes affect species richness with non-heligmosomid helminths?

The overall mean species richness of non-heligmosomid helminths was 0.91 ± 0.029. However, in the presence of heligmosomid nematodes this was higher (heligmosomids present, 1.07 ± 0.042 and absent, 0.70 ± 0.038; GLM with Poisson errors, *Dev*_1,920_ = 35.3, *P* < 0.0001). As the data in Table 5 show, the mean species richness of non-heligmosomid helminths was numerically higher in heligmosomid carrying voles in each of the 4 years of survey, in each of the 3 study sites, in both sexes and all 3 age classes. With both intrinsic and extrinsic factors taken into account this difference was highly significant (GLM with Poisson errors, MSM for non-heligmosomid helminth species richness + P/A heligmosomids, *Dev*_1,906_ = 6.680, *P* = 0.0097), but explained only 0.89% of the variance.

**Table 5.** Mean species richness of non-heligmosomid helminths (i.e. not heligmosomidae) in bank voles with and without heligmosomids by year, site, host sex and age class

		Heligmosomids present		Heligmosomids absent	
Factor	Level	Mean	SEM	Mean	SEM
Year	1999	**0.72**	0.070	0.57	0.120
	2002	**1.42**	0.105	0.82	0.069
	2006	**1.14**	0.071	0.79	0.071
	2010	**1.02**	0.084	0.56	0.065
Site	Urwitałt	**1.11**	0.074	0.74	0.101
	Tałty	**0.99**	0.068	0.74	0.058
	Pilchy	**1.08**	0.068	0.64	0.055
Sex	Males	**0.95**	0.054	0.66	0.052
	Females	**1.20**	0.064	0.74	0.055
Age	Class 1	**0.58**	0.062	0.43	0.042
	Class 2	**0.86**	0.058	0.65	0.058
	Class 3	**1.49**	0.070	1.25	0.094

The higher prevalence at each level is highlighted in bold.

Sample sizes are as in Table 3.

### Does the presence of heligmosomid nematodes affect abundance of other helminths?

Mean abundances of all the different taxa, in voles that were infected or not infected with heligmosomid nematodes, are shown in Table 2. In each case, mean worm burdens were numerically higher in voles that were infected with heligmosomids. Analysis of all the non-heligmosomid helminths, with all significant intrinsic and extrinsic factors and their interactions taken into account, showed that this difference between voles with and without heligmosomids was significant (GLM with negbin errors, FFM for non-heligmosomid helminths + P/A of heligmosomids, *LR*_1,850_ = 6.228, *P* = 0.0126), explaining 0.12% of the variance. As the summary data in Table 6 show, mean worm burdens were numerically higher in voles with heligmosomids in each of the 4 surveys, in each of the 3 study sites, in both sexes and in each of the 3 age classes.

**Table 6.** Abundance of non-heligmosomid helminths (i.e. not Heligmosomidae) and cestodes (all stages combined) in bank voles with and without heligmosomids by year, site, host sex and age class

		Non-heligmosomid helminths				All cestodes			
		Heligmosomids present		Heligmosomids absent		Heligmosomids present		Heligmosomids absent	
Factor	Level	Mean	SEM	Mean	SEM	Mean	SEM	Mean	SEM
Year	1999	**87.2**	48.50	29.7	16.80	**1.53**	0.999	0.14	0.135
	2002	**17.9**	6.21	8.3	3.25	**10.48**	5.470	1.00	0.382
	2006	**23.7**	6.55	14.3	2.82	**8.01**	5.833	0.92	0.653
	2010	**23.3**	4.69	5.5	1.52	**13.01**	4.239	0.89	0.715
Site	Urwitałt	**34.1**	18.33	9.6	4.90	**8.17**	2.445	2.04	0.957
	Tałty	**64.4**	23.73	17.0	4.43	**17.02**	8.410	0.87	0.635
	Pilchy	**12.3**	2.44	5.6	1.18	**2.39**	1.684	0.29	0.170
Sex	Males	**33.7**	16.15	9.9	2.47	**7.74**	3.755	0.86	0.367
	Females	**36.8**	11.84	11.8	3.28	**9.64**	3.200	0.87	0.528
Age	Class 1	**14.3**	4.38	6.2	1.64	**2.63**	2.428	0.73	0.573
	Class 2	**27.3**	15.58	10.7	3.48	**2.17**	0.918	0.73	0.493
	Class 3	**52.1**	19.30	19.4	6.39	**17.20**	5.594	1.31	0.509

The higher abundance at each level is highlighted in bold. Sample sizes are as in Table 3.

When the analysis was confined to non-heligmosomid nematodes, the difference in abundance between voles with or without heligmosomids was also highly significant (GLM with negbin errors, P/A of heligmosomids, *LR*_1,920_ = 29.68, *P* < 0.0001), but lost significance when all the intrinsic and extrinsic factors and their interactions were taken into account (GLM with negbin errors, FFM for non-heligmosomid nematodes + P/A of heligmosomids, *LR*_1,850_ = 2.191, *P* = 0.139; Table S1).

Additional refinement of the analysis, confined to oxyuroid nematodes, showed that there was a highly significant effect of P/A of heligmosomid nematodes on the abundance of oxyuroid nematodes (GLM with negbin errors, P/A of heligmosomids, *LR*_1,920_ = 22.26, *P* < 0.0001), accounting for 0.51% of variance. However, the full factorial model did not converge. A model with all 4 factors as main effects and with 2 interactions (SITE × YEAR and SEX × AGE) plus P/A heligmosomids converged but was marginally non-significant (GLM with negbin errors, BFM for oxyuroid nematodes + P/A of heligmosomids, *LR*_1,904_ = 3.505, *P* = 0.061). A model without SEX, but with all 3 remaining main effects (YEAR, SITE and AGE) and three 2-way interactions (GLM with negbin errors, BFM for oxyuroid nematodes + P/A of heligmosomids, *LR*_1,897_ = 3.928, *P* = 0.047) converged successfully but accounted for only 0.1% of variance. These results suggest a weak effect, after controlling for the intrinsic and extrinsic factors and the interactions that were taken into account. The summary data in Table S1 show that with 1 exception (in the year 2002), mean oxyuroid worm burdens were numerically higher in heligmosomid-infected hosts in other years (1999, 2006 and 2010), in all 3 sites, both sexes and all age classes.

We selected 2 of the nematode species that showed a prevalence exceeding 10% for analysis. The most prevalent oxyuroid nematode was *A. tianjinensis* (Table 1), and as above, without controlling for confounding factors, abundance was higher in voles with heligmosomid nematodes (Table 2; GLM with negbin errors, P/A of heligmosomids on abundance of *A. tianjinensis*, *LR*_1,920_ = 4.33, *P* = 0.0374). Again, FFM for *A. tianjinensis* would not converge, however a model with YEAR + SITE + AGE + all three 2-way interactions did (GLM with negbin errors, BFM for *A. tianjinensis* + P/A of heligmosomids, *LR*_1,897_ = 10.377, *P* = 0.0013, *R*^2^ = 0.28%; see Table S2 for breakdown by main effects and levels within).

The abundance of *M. muris* was also numerically higher in voles concurrently harbouring heligmosomids (Table 2), but the difference was not significant when we fitted a GLM with negbin errors without controlling for intrinsic and extrinsic factors (*LR*_1,920_ = 1.577, *P* = 0.209). Full factorial models for *M. muris* also did not converge but a model comprising all 4 main effects and 5 of the six 2-way interactions (omitting YEAR × AGE) did, revealing that the P/A of heligmosomids was not a significant effect (Table S2; GLM with negbin errors, P/A of heligmosomids, *LR*_1,895_ = 2.212, *P* = 0.137, *R*^2^ = 0.188%). Although, in 8 of the data subsets (Table S2) abundance was numerically higher in heligmosomid carrying voles, in 4, abundance was higher in those without concurrent heligmosomids.

The mean abundance of cestodes (all species combined) was numerically 10 times higher in voles with heligmosomids (Table 2), and without controlling for the intrinsic and extrinsic factors this was a highly significant difference (GLM with negbin errors, P/A of heligmosomids on abundance of cestodes, *LR*_1,920_ = 46.39, *P* < 0.00001). However, models controlling for all possible confounding factors proved highly problematic. The most complex acceptable model was a GLM with negbin errors, SITE × YEAR × AGE and P/A of heligmosomids on abundance of cestodes (*LR*_1,885_ = 14.39, *P* = 0.00015, *R*^2^ = 0.773%). As the data in Table 6 show, abundance was numerically higher in voles also carrying heligmosomids in all 4 surveys, all 3 sites, in both sexes and in all 3 classes. The abundance of intestinal stages of cestodes and that of larval (intermediate stages) cestodes was also higher overall in heligmosomid carrying voles (Table 2; twice and 12 times higher, respectively), and in both cases significantly higher without controlling for confounding factors (GLMs with negbin errors and P/A of heligmosomids on abundance, *LR*_1,920_ = 9.95, *P* = 0.0016 and *LR*_1,920_ = 14.108, *P* = 0.00017, respectively). In voles concurrently infected with heligmosomids, mean abundances were numerically higher in all data subsets corresponding to the main effects (Table 7), however, full factorial models were problematic in each case. For intestinal cestodes, a BFM (comprising all four main effects + five 2-way interactions [excluding YEAR × AGE] and two 3-way interactions (SITE × YEAR × SEX and SITE × SEX × AGE) + P/A of heligmosomids on abundance, *LR*_1,885_ = 0.885, *P* = 0.35, *R*^2^ = 0.084%), indicated that the difference in abundance of intestinal cestodes in voles with and without heligmosomids was not significant. For larval cestodes only a simple model (SITE × AGE + P/A heligmosomids, *LR*_1,912_ = 6.118, *P* = 0.0134, *R*^2^ = 0.634%) converged and indicated a significant effect of the P/A of heligmosomid nematodes on abundance of larval cestodes, but must be interpreted with caution because neither YEAR, nor SEX were taken into account.

**Table 7.** Abundance of intestinal and larval cestodes in bank voles with and without heligmosomids by year, site, host sex and age class

		Intestinal cestodes				Larval cestodes			
		Heligmosomids present		Heligmosomids absent		Heligmosomids present		Heligmosomids absent	
Factor	Level	Mean	SEM	Mean	SEM	Mean	SEM	Mean	SEM
Year	1999	**0.15**	0.045	0	0	**1.38**	1.000	0.14	0.135
	2002	**1.15**	0.265	0.30	0.085	**9.33**	5.453	0.70	0.370
	2006	**0.34**	0.063	0.28	0.137	**7.68**	5.836	0.64	0.642
	2010	**0.28**	0.087	0.16	0.049	**12.73**	4.232	0.73	0.714
Site	Urwitałt	**0.64**	0.116	0.59	0.207	**7.52**	2.441	1.45	0.939
	Tałty	**0.36**	0.106	0.18	0.041	**16.67**	8.406	0.70	0.635
	Pilchy	**0.18**	0.056	0.08	0.034	**2.20**	1.685	0.21	0.167
Sex	Males	**0.50**	0.079	0.24	0.074	**7.23**	3.750	0.61	0.359
	Females	**0.35**	0.091	0.19	0.058	**9.29**	3.201	0.68	0.526
Age	Class 1	**0.18**	0.069	0.15	0.058	**2.45**	2.428	0.57	0.571
	Class 2	**0.25**	0.049	0.17	0.051	**1.92**	0.919	0.56	0.492
	Class 3	**0.70**	0.129	0.42	0.154	**16.50**	5.592	0.90	0.484

The higher abundance at each level is highlighted in bold.

Sample sizes are as in Table 3.

### Does the increasing intensity of heligmosomid burdens covary with increasing species richness of non-heligmosomid helminths?

**Figure 3.**
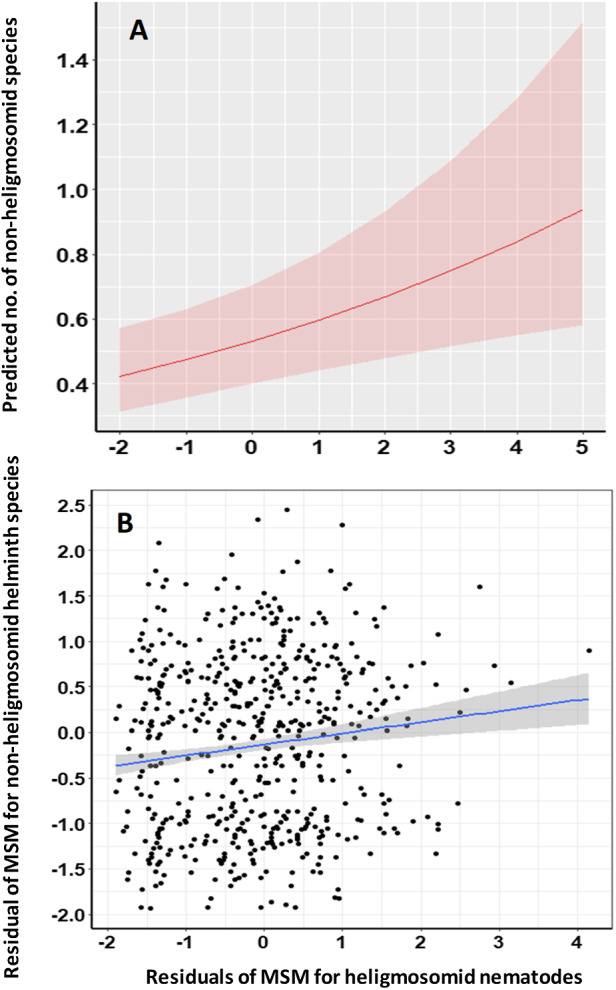
Covariance of the residuals of the Minimum Sufficient Model (MSM) for heligmosomid worm burdens with the residuals of the MSM for non-heligmosomid helminth species richness. Panel A illustrates the predictions of the model in Table 8, whereas panel B shows the regression (*R*^2^ = 0.0148). The shaded areas show the 95% confidence region. For additional details, see text and Table 9.

To control for the possible confounding effects of intrinsic and extrinsic factors known to affect heligmosomid abundance and non-heligmosomid species richness, covariance between these was tested in the MSM for non-heligmosomid helminth species richness either combined with or without the residuals of the MSM for heligmosomids as a covariate. The residuals of the MSM for the heligmosomid burden were a highly significant explanatory term in this model (Table 8, *R*^2^ = 1.32%) with a positive gradient (Figure 3A).

**Table 8.** Quantitative covariance of non-heligmosomid species richness and worm burdens of non-heligmosomid helminth taxa with worm burdens of the heligmosomid nematodes. Models comprised the residuals of the minimum sufficient model (MSM) for heligmosomid burdens as a covariate fitted as an additional explanatory factor to full factorial (FFM), minimum sufficient (MSM) or best-fit (BFM) models of the specified taxa

Taxon	Model^a^	Statistic and value^b^	*P*^2^	Slope (ß) ±	SEM
Species richness^c^	MSM + heligmosomid WB	*Dev*_1,906_ 9.842	0.00085	0.114	0.0359
Non-heligmosomid helminths	FFM + heligmosomid WB	*LR*_1, 850_ 7.833	0.0026	0.2280	0.0705
Non-heligmosomid nematodes	FFM + heligmosomid WB	*LR*_1, 850_ 5.109	0.0119	0.1798	0.0735
Oxyuroidea	BFM + heligmosomid WB	*LR*_1,904_ 2.4227	0.0598	0.1464	0.0937
All cestodes	BFM + heligmosomid WB	*LR*_1, 885_ 11.596	0.00033	0.7525	0.1385
Intestinal cestodes	BFM + heligmosomid WB	*LR*_1,885_ 2.4210	0.0598	0.1598	0.0978
Larval cestodes	BFM + heligmosomid WB	*LR*_1, 912_ 3.8626	0.0247	0.8310	0.2872

a In each case, we first either fitted full factorial, minimum sufficient or best-fit models, derived for the specified taxon from full factorial models (YEAR × SITE × AGE × SEX), and then added the residuals of the minimum sufficient model for heligmosomid worm burdens (WB) as a covariate.

b Test statistic (*LR*, likelihood ratio, distributed as chi-squared or *Dev*, deviance) with degrees of freedom, its value and *P* for comparison of models with and without heligmosomid WB (covariate). *P*-values are 1-tailed since we had predicted positive covariance in all cases.

c Species richness of non-heligmosomid helminths. For this dependent variable, the MSM was based on Poisson errors. For all the other dependent variables, models were based on negative binomial error structures.

Additionally, we also tested the linear relationships between the residuals of the MSMs for non-heligmosomid helminth species richness and those of the MSM for heligmosomid burdens. This relationship was positive (Table 9 and Figure 3B, *r* = 0.126) and highly significant (*t*_920_ = 3.854, *P* = 0.00006), accounting for 1.48% of the variance.

**Table 9.** Quantitative covariance of non-heligmosomid species richness and worm burdens of non-heligmosomid helminth taxa with heligmosomid worm burdens, as reflected in the linear regressions between the residuals of full-factorial, best-fit or minimum sufficient models (as in Table 8). The explanatory variable in all models is the residuals of the MSM for heligmosomid worm burdens

Dependent variable^a^	Adjusted *R*^2^^b^	Slope (*ß*)	± SEM	*t*^c^	DoF^c^	*P*^c^
Species richness^d^	0.0148	0.1208	0.0314	3.854	920	0.00006
Non-heligmosomid helminths	0.0111	0.1031	0.0306	3.373	920	0.00039
Non-heligmosomid nematodes	0.0090	0.0904	0.0300	3.060	920	0.00114
Oxyuroidea	0.0027	0.0474	0.0254	1.866	920	0.0312
Combined cestodes	0.0039	0.0395	0.0184	2.145	920	0.0161
Intestinal cestodes	0.0011	0.0311	0.0220	1.413	920	0.0790
Larval cestodes	0.0015	0.0137	0.0089	1.541	920	0.0619

a The sample size for all = 922.

b The *R*^2^ is for the regression, followed by the gradient of the slope and the SEM of this estimate.

c Student’s *t,* degrees of freedom (DoF) and *P*-values (1-tailed) for test of whether the slopes differ from zero, since our prediction in all cases was for a positive slope.

d Species richness of non-heligmosomid helminths. For this dependent variable the MSM was based on Poisson errors. All the others were based on models with negative binomial error structures.

### Does the increasing intensity of heligmosomid burdens covary with increasing intensities of non-heligmosomid helminth taxa?

**Figure 4.**
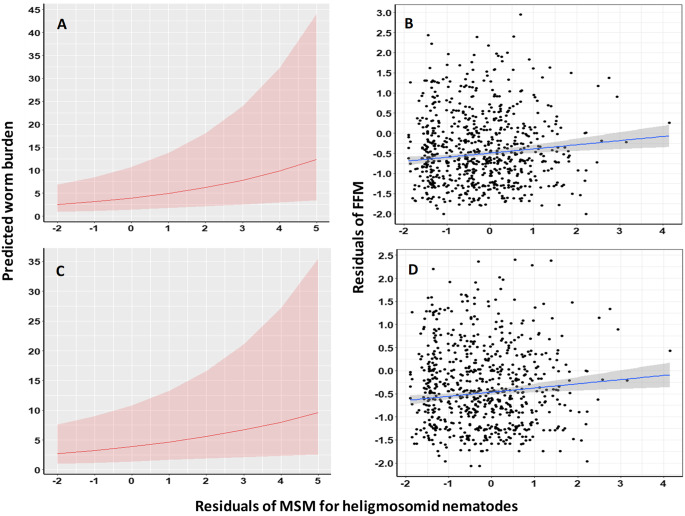
Covariance of the residuals of the Minimum Sufficient Model (MSM) for the heligmosomid burdens with the residuals of Full Factorial Models (FFM) for all non-heligmosomid helminths (A and B) and non-heligmosomid nematode (C and D) worm burdens. The panels on the left (A and C) illustrate the predictions of the relevant models in Table 8, whereas those on the right (B and D) show regressions with FFMs of non-heligmosomid helminth (B; adjusted *R*^2^ = 0.0111) and non-heligmosomid nematode (C; *R*^2^ = 0.0090) worm burdens. The shaded areas show the 95% confidence region. For additional details, see text and Table 9.

The same approach was implemented to assess the degree of covariance of heligmosomid burdens with non-heligmosomid helminth, non-heligmosomid nematode, oxyuroid nematode, combined cestode, intestinal and larval cestode worm burdens. In all cases, these relationships were positive and significant (Tables 8; Figure 4), except in the case of oxyuroid nematodes and intestinal cestodes where the relationships were marginally non-significant.

In linear models testing covariance between the residuals of best-fit models of these taxa and the residuals of the MSM for heligmosomid nematodes, positive slopes were obtained in all cases (Table 9). However, highly significant covariance was found only for non-heligmosomid helminths (*R*^2^ = 1.11%; Figure 4A,B) and non-heligmosomid nematodes (*R*^2^ = 0.9%; Figure 4C,D), with weaker but significant relationships for the Oxyuroidea (*R*^2^ = 0.27%) and combined cestode worm burden (*R*^2^ = 0.39%) evident. As above, the relationships for intestinal cestodes (*R*^2^ = 0.11%) was non-significant, but using this approach, that for larval cestodes (*R*^2^ = 0.15%) also failed to achieve significance.

The database contained multiple cases of animals that were not infected with specific taxa (see Table 1), and because concentrations of double negatives can generate false positive slopes in models of covariance, we repeated the analysis of covariance of residuals from models of relevant taxa, but this time selecting only voles that were infected with at least 1 heligmosomid worm and had at least 1 worm of the specified taxon. Two examples are illustrated in Figure S2 with statistical analyses provided in the figure legend. Significant positive covariance was found for non-heligmosomid helminths and non-heligmosomid nematodes (Figure S2A,B). For intestinal cestodes (*n* = 107, *r* = 0.140, *ß* = 0.153 ± 0.1055, *t*_105_ = 1.447, *P* = 0.0754, Adjusted *R*^2^ = 0.0102) and for *A. tianjinensis n* = 231, *ß* = 0.090 ± 0.0609, *t*_229_ = 1.479, *P* = 0.0703, Adjusted *R*^2^ = 0.0051) covariance was close to significance, but for all cestodes and larval cestodes covariances were not significant. With the exception of the model for larval cestodes, all revealed positive slopes, but none explained more than 0.9% of variance.

In a further approach, the covariance distribution test of Haukisalmi and Henttonen (1998) was applied to the data. All the covariances of the specified taxa with heligmosomid worm burdens (Table 10) were positive and most retained significance when subset grouping (the 4 factors) was taken into account, although *P* values were generally lower. However, covariance of heligmosomids with intestinal cestodes, and with the most prevalent species, *C. henttoneni*, lost significance when we controlled for the 4 factors (subsample constraints).

**Table 10.** Covariance of heligmosomid worm burdens with other taxa, as detected by the covariance distribution test of Haukisalmi and Henttonen (1998)

	Heligmosomid worm burdens					
	Log10 (*x* + 1) transformed			Square root transformed		
		Subsample constraints^a^			Subsample constraints	
Taxon	Cov^b^	Without^c^	With^c^	Cov^b^	Without^c^	With^c^
Non-heligmosomid helminths	0.2343	<0.0001	<0.0001	0.6154	<0.001	0.0030
Non-heligmosomid nematodes	0.1731	<0.0001	0.0018	0.4105	0.0020	0.0198
Oxyuroidea	0.0986	0.0050	0.0100	0.2492	0.0454	0.0438
*Mastophorus muris*	0.0799	<0.0001	0.0060	0.1500	<0.0001	0.0054
*Aspiculuris tianjinensis*	0.0969	0.0042	0.0070	0.2181	0.0064	0.0114
All cestodes	0.1028	<0.0001	0.0120	0.2734	0.0008	0.0196
Intestinal cestodes	0.0316	0.0032	0.1268	0.0584	0.0026	0.0894
Larval cestodes	0.0785	0.0006	0.0264	0.2273	0.0034	0.0490
*Catenotaenia henttoneni*	0.0307	0.0040	0.0940	0.0569	0.0026	0.0672

a Significance (*P* values) of the test on data without subsamples being taken into account, and with subsample constraints taken into account (i.e. with YEAR, SITE, SEX and AGE accounted for). Probability was calculated by comparison of observed data to 5000 randomizations of the data.

b Covariance, as calculated in the covariance distribution test of Haukisalmi and Henttonen (1998).

c Probability (P).

## Discussion

In this paper, we have focused specifically on heligmosomid nematodes and the consequences of their presence for other species of helminths concurrently residing in bank voles. Our study therefore differs from many others seeking evidence for interactions between helminth species in wild caught rodents because our study was based on a hypothesis derived from laboratory experimental studies, and on its clear predictions.

In Western Europe, bank voles are typically subject to infection by 2 closely related heligmosomid species, *Heligmosomum mixtum* and *Heligmosomoides glareoli* (Haukisalmi and Henttonen, 1993a, 1993b; Grzybek et al., 2015), and although these are classified in different genera within the family Heligmosomidae, both on morphological grounds and genetically, they are closely related (Durette-Desset, 1985; Zaleśny et al., 2014; Harris et al., 2015). They are also closely related to *H. bakeri*, a species that has been used as a laboratory model of chronic nematode infections for over 60 years and to *H. polygyrus*, a parasite of wood mice in the W. Palearctic (Durette-Desset, 1985; Zaleśny et al., 2014; Harris et al., 2015), that also gives rise to long-lasting chronic infections in its host (Gregory et al., 1990). Given the now well-established immunomodulatory properties of *H. bakeri* (Behnke et al., 1983; Hewitson et al., 2011; Maizels et al., 2012; Zhang et al., 2023; Lynch and Grencis, 2025), and evidence that the presence of *H. polygyrus* in wood mice is associated with increased species richness of other helminths (Behnke et al., 2005, 2009), we hypothesized that bank voles carrying heligmosomid nematodes should also be subjected to depressed intestinal resistance to infections arising from the long-term survival strategy of these nematodes. On this basis, we predicted that heligmosomid-infected bank voles are more likely to also carry other species of helminths and heavier infections with the latter, compared to bank voles without current heligmosomid infections. Moreover, we expected to see positive covariance between appropriately adjusted heligmosomid worm burdens (i.e. with both intrinsic and extrinsic factors taken into account) and those of other taxa. All these predictions are clearly supported by our results and associated data analysis.

The strongest support for our hypotheses was evident when we examined the relationships of heligmososmid nematodes with all other helminths, i.e. at the highest taxonomic level for the latter, the non-heligmosomid taxon with the highest prevalence in bank voles (62.9%). In heligmosomid carrying bank voles, prevalence of non-heligmosomid helminths was significantly higher (although marginally context dependent), abundance of non-heligmosomid helminths was significantly higher and evidence for quantitative covariance was found in GLMs of the former with heligmosomid worm burdens (fitted as a covariate), in linear regressions of both taxa, after control for intrinsic and extrinsic factors, and in the covariance distribution test, in which context was controlled for (i.e. subset constraints). Moreover, adjusted worm burdens covaried positively and significantly, when the analyses were confined to voles that carried at least 1 specimen of each taxon. Much the same was found when the analyses were confined to non-heligmosomid nematodes (essentially 5 species combined in this taxon, with a collective prevalence of 54.6%; see Table 1), although the relationships were weaker, and the abundance of non-heligmosomid nematodes did not achieve significance when intrinsic and extrinsic factors were taken into account.

The prevalence and abundance of cestodes (all species combined) was also significantly higher in heligmosomid infected bank voles in all the categories of analysis that were undertaken, despite the much lower prevalence of 20.6%. However, quantitative analyses based on GLMs, all proved difficult to implement and we resorted to fitting best-fit reduced models. None of the tests yielded significance for intestinal strobilate worms, for which there was a high preponderance of animals carrying just a single worm (single worm infections were recorded in 95 of the 150 bank voles infected with intestinal cestodes).

In none of our models for non-heligmosomid taxa, even those showing the highest level of significance, did the presence of heligmosomid nematodes account for more than 1.0% of the variance in other taxa. The most marked effects were evident when we fitted species richness of non-heligmosomid helminths (i.e. by summing the number of species present in bank voles, rather than that of individual worms). For covariance of non-heligmosomid helminth species richness with heligmosomids, and after adjusting for the presence of intrinsic and extrinsic factors, our models accounted for up to 1.48% of variance.

Previous studies have generally found associations of intestinal helminths in rodents to be difficult to detect, rare and mostly context dependent. With respect to heligmosomids, Mongomery and Montgomery (1990) found significant positive but mostly weak co-occurrence of *H. polygyrus* (referred to as *N. dubius* in their paper) with *Corrigia vitta* and *Syphacia stroma*. Although Lewis et al. (2023) recorded an arithmetically higher prevalence of *C. vitta* in *H. polygyrus*-infected wood mice compared to those without the latter species, this was not a significant difference, and there was no quantitative interaction between these species.

Among the earlier attempts to identify whether specific helminths co-occur together more frequently than not, Kisielewska (1970) found that *H. halli* (but probably more likely *H. glareoli* or *H. mixtum*) co-occurred with *C. pusilla* (*C. henttoneni*) in bank voles in Eastern Poland. Behnke et al. (2005) reported significantly higher prevalence of *C. pusilla* in wood mice carrying *H. polygyrus* compared to those without the latter species, and significant positive correlation of worm burdens, even after controlling for context. However, while Lewis et al. (2023) found numerically higher prevalence of *C. pusilla* in *H. polygyrus*-carrying wood mice compared to those without the latter species, this was not a significant difference when context was not accounted for. With context taken into account, Lewis et al. (2023) found that there was a significant interaction with age; prevalence of *C. pusilla* was similar among *H. polygrus*-infected juvenile (7.9%) and adult mice (9.4%), but among mice without *H. polygrus* prevalence of *C. pusilla* was higher in adults (10.7%) compared to juveniles (0%). Moreover, in analyses confined to mice carrying both *H. polygyrus* and *C. pusilla*, there was a weak positive significant correlation of worm burdens, and this remained significant when the data were controlled for both intrinsic and extrinsic factors.

Another approach used in earlier studies was to assess the effect of *H. polygyrus* on prevalence and abundance of other helminths (i.e. as reflected in combined worm counts and species richness of non-heligmosomid helminths). Behnke et al. (2005) reported significant correlation of *H. polygyrus* worm burdens with species richness of other helminth species both after fitting raw data and data adjusted for context, but not with the total worm burdens of the other species. In a subsequent study based on analysis of 3 independent data sets (2 localities in England and a dataset based on 6 sites in Portugal), Behnke et al. (2009) found much the same, higher prevalence of other helminths and higher non-heligmosomid species richness in mice infected with *H. polygyrus*. In contrast to these studies and the current work, Lewis et al. (2023) found that while prevalence of other helminths (all non-heligmosomids combined) and non-heligmosomid species richness increased with increasing worm burdens of *H. polygyrus*, the significance was lost when the data were adjusted for context.

Where found to be significant in previous studies, associations between helminth species and correlations in quantitative data have generally been found to be weak and often context dependent. This may be partly due to sample sizes that are too small to detect subtle effects, especially in data sub-sets, and the use of inappropriate statistical methods. Moreover, there are many reasons why a process of functional importance might nonetheless appear to be of small effect size in cross-sectional correlations. This could be due to masking by any of the confounding processes arising from correlated susceptibility or correlated exposure considered above (see Introduction). Additionally, the statistical adjustment for variables such as sex or size or season or locality, although highly desirable when attempting to increase the inference that 1 species might influence another (the key interest here), may also partition out a genuine signal that is collinear with the adjusted variable(s). For example, adjustment for size may mask facilitatory interactions, as these might increase in magnitude as hosts age, grow and are exposed to more parasites (with age, size and parasite exposure likely to be collinear to some extent).

Some have argued that conclusions of the occurrence of quantitative interactions among multiple parasite species in host populations may be unreliable when derived from cross-sectional studies at single time points and associated worm burdens at autopsy (Poulin, 2005) and are unlikely to detect interactions accurately (Knowles et al., 2013; Fenton et al., 2014; Sweeny et al., 2021; Barger, 2023). Nevertheless, when similar relationships can be detected in different data subsets, collected at different sites in different years, as in the current work, some confidence in their repeatability and hence robustness is warranted (Behnke et al., 2024). Moreover, where there is consistency with known biological properties (such as the well-known immunosuppressive properties of heligmosomids leading to an expectation of positive interaction and hence positive association) then a stronger causal inference can be sustained.

Haukisalmi and Henttonen (1993a) concluded that helminth infections in bank vole from their study sites in Finland were mostly independently aggregated in hosts, but that rarely occurring species were restricted to hosts with concurrent common species. To some degree our data support this conclusion, both in that all the significant associations and correlations we detected were weak and in that cestodes, the taxon with the lowest prevalence in our data, showed higher prevalence and abundance in heligmosomid infected voles. Lewis et al. (2023) and Behnke et al. (2005) both also concluded that quantitative interactions are weak, context dependent, perhaps reflecting assemblages of helminth species, rather than interacting communities.

Our analyses demonstrate the powerful influence of controlling for both intrinsic and extrinsic factors, since when we failed to fit these explanatory factors, all of our analyses were highly significant. While it is necessary to carry out such adjustments to increase the inference of interactions between parasites, nonetheless we note that this may also be masking genuine signals of interaction, and reducing their observed effect size, when these are collinear with the adjusted variables. Taken together, our analyses support our prediction that bank voles carrying adult heligmosomid worms are likely to be infected also with other helminths, and that to some extent this relationship is quantitative. In no small measure, the large sample size of our dataset enabled the detection of the subtle (confounder-adjusted) effect of heligmosomid nematodes on other concurrently residing helminth taxa. In this respect, our current findings concur with earlier reports seeking evidence for interactions between helminths in wild caught rodents. Behnke et al. (2005) concluded that while associations and interactions between helminths in wood mice in S. England could be detected in cross-sectional surveys, they were highly context dependent and unlikely to play a dominant role among processes involved in structuring helminth component communities in this host species. However, in this study and in a subsequent work analysing data from wood mice from sites in England and Portugal (Behnke et al., 2009), the likelihood of being infected with non-heligmosomid helminth species, as reflected in non-heligmosomid species richness, was more likely in heligmosomid infected mice and a highly predictable element of co-infections in wood mice. Our current results support this view and extend it to infections in bank voles. Indeed, given the consonance of these results in gastrointestinal communities containing heligmosomid species, and similar results observed in spirurid-dominated communities (Behnke et al., 2024), it might be predicted that many vertebrate gastrointestinal communities are structured by immunosuppressive gastrointestinal nematodes that act as ecosystem engineers (Wright et al., 2002), impacting species richness and heterogeneity in community assemblages.

## Supporting information

10.1017/S0031182025101376.sm001 Behnke et al. supplementary material Behnke et al. supplementary material

## Supplementary material

The supplementary material for this article can be found at https://doi.org/10.1017/S0031182025101376.
