# Supplementary material for: Heligmosomid infections in bank voles are associated with higher prevalence and greater abundance of other helminth species
Source: Parasitology. 2025 Dec 12;153(2):198–213. doi: 10.1017/S0031182025101376 (PMC13215745; doi:10.1017/S0031182025101376)
Supplement: Behnke et al. supplementary material [file S0031182025101376sup001.docx]

**Supplementary data**

**Table S1.** Abundance of non-heligmosomid nematodes (i.e. not Heligmosomidae) and oxyuroid nematodes in bank voles with and without heligmosomids by year, site, host sex and age class.

|  |  | Non-heligmosomid nematodes | | | | Oxyuroid nematodes | | | |
| --- | --- | --- | --- | --- | --- | --- | --- | --- | --- |
|  |  | Heligmosomids present | | Heligmosomids absent | | Heligmosomids present | | Heligmosomids absent | |
| Factor | Level | Mean | SEM | Mean | SEM | Mean | SEM | Mean | SEM |
| Year | 999 | **85.7** | 48.52 | 29.6 | 16.80 | **85.4** | 48.52 | 29.6 | 16.80 |
|  | 2002 | **7.4** | 2.98 | 7.3 | 3.23 | 5.7 | 2.96 | **6.6** | 3.22 |
|  | 2006 | **15.7** | 2.94 | 13.3 | 2.74 | **14.8** | 2.93 | 12.2 | 2.72 |
|  | 2010 | **10.3** | 2.04 | 4.6 | 1.31 | **5.6** | 1.54 | 3.8 | 1.21 |
| Site | Urwitałt | **25.9** | 18.19 | 7.5 | 4.78 | **22.9** | 18.18 | 5.6 | 4.73 |
|  | Tałty | **47.4** | 22.42 | 16.2 | 4.40 | **46.4** | 22.43 | 16.0 | 4.40 |
|  | Pilchy | **9.9** | 1.78 | 5.3 | 1.16 | **8.4** | 1.74 | 4.4 | 1.09 |
| Sex | Males | **25.9** | 15.74 | 9.0 | 2.44 | **25.5** | 15.75 | 8.7 | 2.44 |
|  | Females | **27.2** | 11.45 | 10.9 | 3.24 | **23.4** | 11.44 | 9.6 | 3.22 |
| Age | Class 1 | **11.7** | 3.71 | 5.4 | 1.53 | **11.5** | 3.72 | 5.2 | 1.52 |
|  | Class 2 | **25.1** | 15.57 | 10.0 | 3.44 | **24.8** | 15.57 | 9.8 | 3.44 |
|  | Class 3 | **34.9** | 18.56 | 18.1 | 6.39 | **30.4** | 18.57 | 15.4 | 6.37 |

The higher abundance at each level is highlighted in bold. Sample sizes are as in Table 3.

**Table S2.** Abundance of *Aspiculuris tianjinensis* and *Mastophorus muris* in bank voles with and without heligmosomids by year, site, host sex and age class.

|  |  | *Aspiculuris tianjinensis* | | | | *Mastophorus muris* | | | |
| --- | --- | --- | --- | --- | --- | --- | --- | --- | --- |
|  |  | Heligmosomids present | | Heligmosomids absent | | Heligmosomids present | | Heligmosomids absent | |
| Factor | Level | Mean | SEM | Mean | SEM | Mean | SEM | Mean | SEM |
| Year | 1999 | **8.3** | 3.25 | 3.1 | 1.47 | **0.29** | 0.110 | 0.03 | 0.027 |
|  | 2002 | 2.9 | 1.12 | **3.4** | 1.10 | **1.52** | 0.486 | 0.57 | 0.241 |
|  | 2006 | **14.2** | 2.90 | 12.0 | 2.73 | **0.82** | 0.230 | 0.46 | 0.206 |
|  | 2010 | **5.6** | 1.54 | 3.8 | 1.21 | 0.71 | 0.196 | **0.79** | 0347 |
| Site | Urwitałt | **2.8** | 0.91 | 0.4 | 0.13 | 0.75 | 0.154 | **0.95** | 0.449 |
|  | Tałty | **18.0** | 3.98 | 9.8 | 2.01 | **0.04** | 0.023 | 0.02 | 0.014 |
|  | Pilchy | **8.3** | 1.74 | 4.4 | 1.09 | **1.54** | 0.357 | 0.89 | 0.286 |
| Sex | Males | 5.2 | 1.30 | **5.8** | 1.38 | **0.34** | 0.092 | 0.26 | 0.156 |
|  | Females | **11.7** | 2.15 | 5.7 | 1.18 | **1.30** | 0.247 | 0.89 | 0.258 |
| Age | Class 1 | **11.5** | 3.72 | 5.1 | 1.52 | 0.12 | 0.064 | **0.21** | 0.177 |
|  | Class 2 | **5.0** | 1.29 | 4.2 | 1.05 | **0.29** | 0.073 | 0.16 | 0.077 |
|  | Class 3 | **9.9** | 2.04 | 9.1 | 2.25 | 1.59 | 0.292 | **1.81** | 0.523 |

The higher prevalence at each level is highlighted in bold. Sample sizes are as in Table 3.

**Fig. S1.** Frequency distribution of worm burdens with *Heligmosomum mixtum* (A; *I*=4.818, *D*=0.783, *k* = 0.268 ± 0.0004), *Heligmosomoides glareoli* (B; *I*=10.689, *D*=0.899, *k* = 0.093 ± 0.0001) and the two heligmosomids combined (C; *I*=6.103, *D*=0.689, *k* = 0.487 ± 0.0011). The figure shows the observed data (filled columns) and those expected of a negative binomial distribution of each of these datasets (red line).

**Fig. S2.** Covariance of residuals from MSM for heligmosomid worm burdens and those of FFM for specified taxa among voles that were infected with at least one worm of each taxon.

We first fitted the MSM for heligmosomid worms and FFM for non-heligmosomid helminths and non-heligmosomid nematodes and saved the residuals of these models. Then we selected records of voles that had at least one heligmosomid worm and at least one worm of the specified taxon. The figs. show the covariance and the 95% confidence limits.

A. *N*=357, *r*=0.105, *ß* = 0.110 ± 0.0551, *t*_355_=1.994, *P*=0.0235, adjusted *R*^2^ = 0.0083

B. *N*=305, *r*=0.101, *ß* = 0.091 ± 0.0515, *t*_303_=1.774, *P*=0.0331, adjusted *R*^2^ = 0.0070
